# Updates on the Renin–Angiotensin–Aldosterone System and the Cardiovascular Continuum

**DOI:** 10.3390/biomedicines12071582

**Published:** 2024-07-17

**Authors:** Dana Pop, Alexandra Dădârlat-Pop, Raluca Tomoaia, Dumitru Zdrenghea, Bogdan Caloian

**Affiliations:** 1 4th Department of Internal Medicine, Faculty of Medicine, “Iuliu Hațieganu” University of Medicine and Pharmacy, 400347 Cluj-Napoca, Romania; pop67dana@gmail.com (D.P.); raluca.tomoaia@gmail.com (R.T.); dzdrenghea@yahoo.com (D.Z.); bogdan912@yahoo.com (B.C.); 2 Cardiology Department, Rehabilitation Hospital, 400012 Cluj-Napoca, Romania; 3 Cardiology Department, Heart Institute “N. Stăncioiu”, 400001 Cluj-Napoca, Romania

**Keywords:** renin–angiotensin–aldosterone system, cardiovascular risk factors, stroke, heart failure, atrial fibrillation

## Abstract

The cardiovascular continuum describes how several cardiovascular risk factors contribute to the development of atherothrombosis, ischemic heart disease, and peripheral arteriopathy, leading to cardiac and renal failure and ultimately death. Due to its multiple valences, the renin–angiotensin–aldosterone system plays an important role in all stages of the cardiovascular continuum, starting from a cluster of cardiovascular risk factors, and continuing with the development of atherosclerosis thorough various mechanisms, and culminating with heart failure. Therefore, this article aims to analyze how certain components of the renin–angiotensin–aldosterone system (converting enzymes, angiotensin, angiotensin receptors, and aldosterone) are involved in the underlying pathophysiology of the cardiovascular continuum and the possible arrest of its progression.

## 1. Introduction

The cardiovascular continuum was first described by Victor J. Dzau et al. in 2006 [1] as a chain of events precipitated by several cardiovascular risk factors, which may lead to the occurrence of atherothrombosis, ischemic heart disease, and peripheral arteriopathy if left untreated. Also, major complications such as cardiac and renal failure and finally death may occur [1].

The renin–angiotensin–aldosterone system (RAAS) and its significant classical role in the occurrence of arterial hypertension was first discussed in the 1960s [2,3]. In 1976, the first converting enzyme inhibitor, captopril, was patented, and in the early 1980s it was recognized as a medication for lowering blood pressure values [4]. Afterwards, other converting enzyme inhibitors (ACEIs) were developed, and then the blockers of AT1 receptors of angiotensin II (ARBs) or sartans were discovered. Over time, the understanding of RAAS has expanded tremendously, with ACEIs and ARBs having an important role in the treatment of heart failure by blocking RAAS at different steps. Also, these drugs, especially ACEIs, have anti-atherosclerotic effects, too. RAAS is ubiquitous in multiple organ systems, especially the kidneys, systemic vasculature, and adrenal cortex, and is strongly involved in the pathogenesis of hypertension, heart failure, other cardiovascular diseases, and renal diseases. Due to its multiple valences, RAAS plays a crucial role in all stages of the cardiovascular continuum, starting with several cardiovascular risk factors, and continuing with the development and progression of atherosclerosis, ultimately leading to the onset of heart failure.

**Figure 1:**
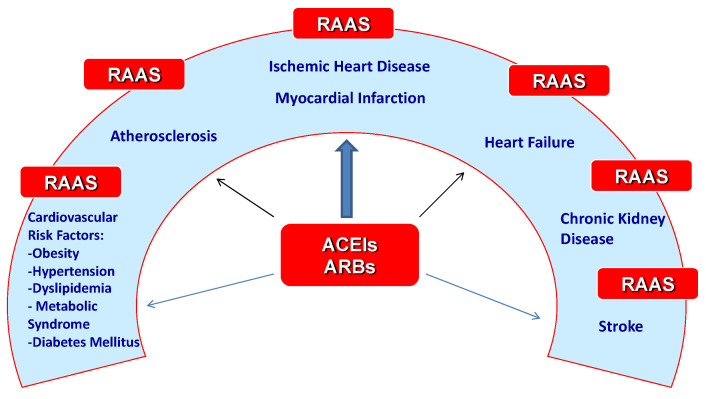
RAAS and the pathogenesis of various conditions within the cardiovascular continuum.

Therefore, the discussion in this article is focused on certain components of the renin–angiotensin–aldosterone system (converting enzymes, angiotensin, angiotensin receptors, and aldosterone) and their involvement in the pathogenesis of various conditions within the cardiovascular continuum, as you can see in Figure 1.

## 2. RAAS and Several Cardiovascular Risk Factors

### 2.1. Hypertension

**Figure 2:**
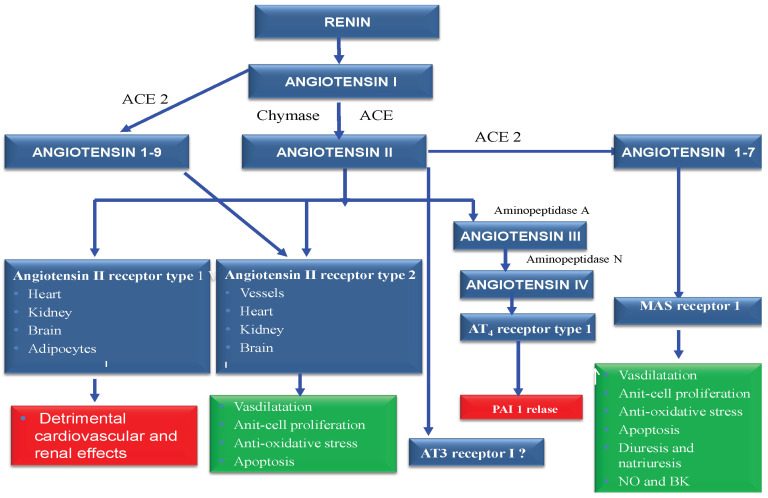
RAAS components—the angiotensins, the converting enzymes and their specific receptors.

The role of RAAS in the pathogenesis of arterial hypertension is already well known. The juxtaglomerular cells favor the cleavage of prorenin to renin. The activation of prorerin in the kidney is stimulated by several enzymes such as proconvertase 1 and cathepsin B [5]. Renin degrades angiotensinogen (AGT) synthesized in the liver into angiotensin I. Cathepsin D and tonins also have the same AGT action [5]. Angiotensin I (AG I) under the action of the “classic” converting enzyme (ACE) is transformed into angiotensin II (AG II)—Figure 2.

However, there are other enzymes called non-EC enzymes (chymase and cathepsin G) that have the same effects [5].

Under pathological conditions, the excess of AG II causes stimulation of AG II AT1 receptors, leading to arterial vasoconstriction, sodium and water retention due to increased release of aldosterone, and finally to atrial remodeling and stimulation of the sympathetic nervous system. All these pathophysiological mechanisms are responsible for arterial hypertension development.

On the other hand, AT2 receptor stimulation results in vasodilatation and nitric oxide release, with proven antiproliferative effects [5]. At the same time, the conversion enzyme determines the degradation of bradykinin (into inactive peptides), which is known as a substance with important vasodilatory effects. The renin–angiotensin-converting enzyme-2 (ACE2) system has a central role in triggering some counter-regulatory mechanisms compared to the classic converting enzyme, with a structure somewhat similar to it (42% of the terminal amino acid structure is identical in the two enzymes).

ACE-2 acts on AG I, converting it to AG (1–9) and on AG II by hydrolyzing it to AG (1–7). Thus, ACE-2 has the role of decreasing the level of AG II where there are increases and activations of this hormone, as you can see in Figure 2.

Thus, EC-2 represents an important counter-regulatory substance of AG II, not only due to the hydrolysis of AG II, but also due to the production of AG (1–7) which, through actions on specific Mas receptors, determines the release of nitric oxide synthase (NOS) and increased sodium excretion—Figure 2. Also, AG (1–7) acts on AT2 receptors and type 2 bradykinin (BK) receptors, which implies important vasodilatory actions. In recent years, several components of the system have been identified, such as AG (1–12), angiotensin A, and alamandin—Figure 2.

Alamandin is produced by the degradation of angiotensin A by ACE-2 or directly from AG (1–7). It acts on specific receptors, such as -MrgD, which also has vasodilatory, antifibrotic, antiproliferative, and natriuretic effects [6,7]. So, we are talking about receptors whose stimulation causes favorable, cardioprotective effects (AT2, Mas, and MrgD) and about AT1 receptors with detrimental cardiovascular effects [8].

In healthy people, there is a balance between the production of ACE and ACE-2. In conditions of arterial hypertension, this balance is lost in favor of the production of ACE [8].

Considering the important role that the renin–angiotensin–aldosterone system plays in the pathogenesis of hypertension, current guidelines recommend angiotensin-converting enzyme inhibitors (ACEIs) or sartans (ARBs) as the first line of treatment in this disease in most patients if there are no contraindications [9].

### 2.2. Obesity

Obesity is another important risk factor often associated with high blood pressure. In people with obesity, a series of substances called adipokines are secreted from within richly represented adipose tissue. These are mainly represented by adiponectin, leptin, omentin, resistin, visfatin, TNF-α, IL-4, CRP, and PAI-1, but also by AGT. Apart from adiponectin and omentin, all other adipokines, and therefore also AGT, are involved in the emergence of endothelial dysfunction [10].

At the same time, the existence of local AG II in adipose tissue and AT1 and AT2 receptors at the adipocyte level have been described [11]. Another interesting thing demonstrated is that stimulation of AT2 receptors by AG II promotes preadipocyte differentiation, and, in humans, AG II generated by mature adipocytes suppresses the differentiation of adipocyte precursors, decreasing the proportion of small insulin-sensitive adipocytes [12]. AGII also has other actions at the adipocyte level: it determines leptin release, increases PGI2 secretion, and favors the activity and transcription rate of glycerol-3-phosphate dehydrogenase and fatty acid synthase [13]. On the other hand, in people with obesity, the presence of increased insulin resistance is accompanied by the amplification of renin activity [13].

The renin–angiotensin system, locally present, plays an important role in the development of adipocytes [13]. At the same time, the presence of important components of the renin–angiotensin system at the adipocyte level could have detrimental cardiovascular effects in people with obesity, which could lead to the creation of a vicious circle of obesity–arterial hypertension–diabetes–atherosclerosis.

### 2.3. Diabetes Mellitus

Diabetes mellitus is frequently associated with hypertension and obesity.

Animal studies have shown that in the presence of prediabetes, there is important activity of the renin–angiotensin system. Thus, increased values of renin, angiotensinogen, ACE, AT1 receptor, aldosterone, and AG II were detected in these studies [14]. The presence of diabetes in the context of hyperglycemia increases insulin resistance and causes endothelial dysfunction with the activation of the renin–angiotensin system and levels of AG II.

The important role of the renin–angiotensin system in diabetes was demonstrated by the evidence provided by ACEIs and ARBs in the treatment of diabetic nephropathy, in which they determined the decrease in proteinuria, renoprotective effects, and the regression of this complication [15]. Moreover, at the renal level, there are all the components of RAAS. The renal effects of the excess release of AG II cause glomerular sclerosis, tubulointerstitial fibrosis, and a reduction in nephron mass with the appearance of chronic nephropathy through multiple mechanisms.

At the retinal level, there are components of the renin–angiotensin system: renin, AGT, AG II, EC, EC-2, AG 1–7, AG II, AT1, AT2, and Mas receptors [16]. AG II has an important role in maintaining local homeostasis, with the control of vasoconstriction, the regulation of glial cell function, and the modulation of neuronal functions [17].

In a study conducted in Canada in which type 1 diabetes patients were included, it was demonstrated that the renin–angiotensin–aldosterone system is involved in the occurrence of diabetic retinopathy [18]. It contributes to the increase in vascular permeability and cell proliferation with important pro-inflammatory effects and increasing oxidative stress, favoring the development of diabetic retinopathy [19].

Under these conditions, ACEIs and ARBs have proven their effectiveness in patients with diabetic retinopathy. Thus, lisinopril administered to patients with type 1 diabetes with normal blood pressure caused a decrease in the progression of diabetic retinopathy of up to 50% [20]. At the same time, the administration of candesartan in patients with type 2 diabetes was associated with a decrease in the progression of retinopathy [21].

### 2.4. Dyslipidemia

The accumulation of Ox-LDL in the arteries causes the activation of the local RAAS, which further contributes to the production of LDL and its oxidation into ox-LDL. All this leads to increased oxidative stress and inflammation [22,23]. At the same time, there is recent evidence showing that megalin, an important component of the low-density lipoprotein receptor superfamily, can contribute to RASS activation, promoting atherosclerosis [24].

### 2.5. Obstructive Sleep Apnea

Recent data highlight a possible role of RASS in obstructive sleep apnea. Thus, in a meta-analysis published in 2023 that includes data collected from 20 studies that included 2828 participants, it is shown that patients with obstructive sleep apnea not only have higher levels of the components of the renin–angiotensin–aldosterone system, but also have higher values of blood pressure and heart rate compared to those without this pathology, even among patients without treatment-resistant hypertension [25].

## 3. Renin–Angiotensin–Aldosterone System and Renal Function

Excessive production of AG II and aldosterone in pathological situations is associated with the development and progression of kidney diseases, such as diabetic or non-diabetic nephropathy. The mechanisms involved in this sense would be increased glomerular capillary pressure, profibrotic effects, and proteinuria [26].

The damage to the nephrons in the context of some pathological renal injuries causes the increase in filtration and the increase in glomerular capillary pressure, with consequent damage to the glomeruli. RASS is involved in the synthesis of nephrin, a protein that is formed at the transmembrane level, in glomerular podocytes [27] which has a role in limiting the release of proteins from the glomerular level.

Activation of RASS causes a decrease in the release of nephrin with the appearance of proteinuria. AG (1–7) determines, at the renal level, the stimulation of phospholipase activity, the stimulation of sodium transport in the proximal tubule, natriuresis, diuretics, and the increase in glomerular filtration [28].

At the renal and local levels, there are components of RASS. AG II is involved in cell proliferation, increased collagen deposits, and apoptosis, and the appearance of glomerular sclerosis and tubulointerstitial fibrosis, all of which lead to glomerular sclerosis and tubulointerstitial fibrosis. Further evolution towards reducing the number of nephrons and chronic nephropathy is needed.

Stimulation of AT1 receptors at the renal level causes local vasoconstriction, increased renal sodium reabsorption, activation of pro-inflammatory cytokines, activation of oxidative stress and endothelial dysfunction, increased PAI-1 activity, and promotion of thrombosis [29]. On the other hand, stimulation of renal AT2 receptors leads to renal and systemic vasodilatation, a decrease in renal sodium reabsorption, inflammation, mitogenesis, and fibrosis [29].

RASS blockers have renoprotective effects, independent of blood pressure values, and can prevent proteinuria and its regression when it exists. These medications cause vasodilatation of the related arterioles with a reduction in intraglomerular pressure, regression of remodeling at the arterial level, and improvement of endothelial function at the level of resistance arterioles, especially in patients with nephrosclerosis [29].

## 4. Implications of the Renin–Angiotensin–Aldosterone System in the Development of Atherosclerosis

The presence of the risk factors mentioned above can contribute over time to the appearance of the atherosclerosis process. The RAAS is also involved in the generation and progression of the atheroma plaque. RAAS components act on all cells involved in plaque formation at the level of smooth muscle cells, endothelial cells, and macrophages [30].

At the same time, AT1, AT2, and Mas receptors exist at these levels [31]. Through the actions of AG II and AG IV, endothelial dysfunction is promoted. The RAAS plays a key role in regulating the inflammatory response by attracting inflammatory cells to the site of arterial wall insult. At the same time, local synthesis of RAAS components takes place at the level of inflammatory cells [32]. Other mechanisms by which AG II can contribute to the formation of atheroma plaque are represented by vasoconstriction, endothelial dysfunction, bradykinin degradation, effects on fibrinolysis, increased release of nitric oxide, and plasminogen activator inhibitor I.

On the contrary, bradykinin and angiotensin (1–7) contribute to maintaining a healthy endothelium [32]. Thus, it is clear that ACEs can have anti-atherosclerotic effects. Several ACEIs have proven cardioprotective effects due to their effects on the bradykinin cascade, fibrinolysis, and the decrease in the levels of AG II [33].

In the HOPE trial, patients with high cardiovascular risk were included, and were administered ramipril. Authors reported a decrease in the rate of occurrence of cardiovascular mortality, acute myocardial infarction, or stroke. The beneficial actions of ramipril were mostly independent of the reduction in blood pressure values, and also of the presence of left ventricular hypertrophy, which argues for the direct anti-ischemic effects of ramipril [34].

Treatment with perindopril in the EUROPA study, in patients with stable angina pectoris and low cardiovascular risk, led to a significant decrease in the main objectives represented by cardiovascular mortality, myocardial infarction, and cardiovascular resuscitations [35]. In the PERSPECTIVE sub-study within the EUROPA trial, the same ACE achieved more than that, namely, stabilization of the coronary plaque evaluated by intravascular ultrasonography [36].

In another study with perindopril, PREAMI, patients over 65 years of age with myocardial infarction and normal ejection fraction were evaluated, and administration of this ACE led to a significant decrease in the risk of heart failure and death [37]. In the PERTINENT study, the effects of perindopril on some markers of atherosclerosis were followed. Its administration caused significant decreases in the levels of angiotensin II, tumor necrosis factor-alpha, von Willebrand factor, and increased levels of nitric oxide, bradykinin, and nitrates/nitrites [38], but did not influence the levels of C-reactive protein and fibrinogen [39].

The addition of perindopril to amlodipine, in the ASCOT-BPLA study, in hypertensive patients determined a significant reduction in the rate of cardiovascular events, the risk of nonfatal myocardial infarction, and fatal ischemic heart disease compared to “standard” β-blocker (atenolol) antihypertensive therapy ± diuretic (thiazide) [40]. This reduction has been observed since the introduction of perindopril therapy [40].

Anti-atherosclerotic effects were also proven by quinapril therapy, which, when administered to post-PTCA patients, caused a significant reduction in the rate of restenosis compared to placebo in the TREND study [41].

The benefits of ACE inhibitor therapy in myocardial infarction were primarily demonstrated by the SMILE trial, in which administration of zofenopril within 24 h of the onset of the myocardial infarction resulted in a significant reduction in mortality after 6 weeks of treatment, which was maintained or increased after one year of treatment [42].

A meta-analysis published in 2000 that included three large trials (SAVE, TRACE, AIRE) concluded that ACE inhibitors administered 48 h from the onset of AMI caused a decrease in mortality, a reduction in the risk of heart failure and the number of hospitalizations for this condition. At the same time, there was also a reduction in the risk of reinfarction [43]. In conclusion, the trials that included patients with various forms of ischemic heart disease and who benefited from ACEI therapy (SAVE, AIRE, TRACE, SMILE, EUROPA, and QUIET) demonstrated the benefits of this class of drugs in terms of reducing cardiovascular events.

The anti-atherosclerotic effects of ARBs are controversial. Undoubtedly, ARBs also have anti-inflammatory effects, demonstrated in animal studies, that are largely mediated by blocking AT1 receptors, inhibiting the release of pro-inflammatory cytokines, such as tumor necrosis factor (TNF)-α, interleukin (IL)-6, and decreased aldosterone release [44,45]. ARBs do not cause an increase in BK synthesis and contribute to an increase in AG II levels.

Under these conditions, AG II actions are not exerted on AT1 receptors, but can be exerted on alternative pathways with the production of AG III and IV with overactivation of AT2 and AT4 receptors [33]. Stimulation of AT2 receptors by AG II leads to the release of prostaglandin E2, which mediates the release of matrix metalloproteinases from macrophages, an enzyme with an important role in the rupture of the atheroma plaque [46].

In turn, AGIV, by stimulating AT4 receptors, causes an increase in the production of nuclear factor-κB and the release of other pro-inflammatory factors that act on vascular smooth muscle cells [44,47]. Blocking AT4 receptors, however, also causes important myocardial antiapoptotic, antifibrotic, and antihypertrophic effects [44,48]. The TRANSCEND study (Telmisartan Randomized Assessment Study in ACE-I Intolerant Subjects with Cardiovascular Disease) evaluated the role of telmisartan in patients diagnosed with ischemic heart disease or diabetes with organ damage, who did not tolerate ACE inhibitors. Authors reported a significant reduction in the risk of major cardiovascular events [49]. At the same time, in the ONTARGET study (Ongoing Telmisartan Alone and in Combination with Ramipril Global End-point Trial) the effectiveness of telmisartan was similar to that of ramipril in reducing the risk of cardiovascular mortality, myocardial infarction, stroke, or hospitalizations for heart failure. It was also evaluated when it was administered to patients with vascular diseases or diabetics with increased cardiovascular risk, without heart failure [50]. Another ARB, losartan, had similar effects to captopril in post-myocardial infarction patients [51].

In the OLIVUS study (Impact of OLmesartan on progression of coronary atherosclerosis: evaluation by IntraVascular UltraSound), the administration of olmesartan to some patients with stable angina pectoris caused a decrease in the volume of the atheroma plaque [52].

Among the ARBs, only valsartan proved its effectiveness in reducing cardiovascular events after myocardial infarction [53], with the VALIANT study demonstrating that valsartan administered post-myocardial infarction causes the same decrease in mortality as captopril [53].

ACEIs (or ARBs) are indicated in the European ESC for the secondary prevention of ischemic heart disease in patients who also have heart failure, diabetes, or arterial hypertension [54].

Aldosterone-related medication has important antifibrotic actions, including the regression of myocardial remodeling and myocardial infarction. In the EPHESUS study, which included post-myocardial infarction patients with an ejection fraction of less than or equal to 40%, patients were given 50 mg of eplerenone daily, and there was a significant decrease in all-cause mortality, cardiovascular mortality, and hospitalizations for heart failure [55]. The same results were obtained in the RALES study performed with spironolactone [56]. The ESC guidelines recommend anti-aldosterone medication in patients with a history of myocardial infarction, with EF ≤40%, with heart failure or diabetes, and without renal failure or hyperkalemia, along with treatment with ACE inhibitors and beta-blockers [54].

## 5. The Renin–Angiotensin–Aldosterone System and Stroke

The excess secretion of AG II also has negative effects on cerebral vessels. It increases inflammation and promotes atherosclerosis at this level, activates the sympathetic nervous system and also the local RAAS. This is because, at the cerebral level, there is also a component of the RASS, including AT1 and AT2 receptors [57].

Both ACEIs and ARBs have proven their effectiveness in the secondary prevention of cerebral vascular accidents [58]. Thus, in the PROGRESS (the perindopril protection against recurrent stroke) study, perindopril was determined to reduce the risk of recurrent stroke, with the benefits being similar in both normotensive and hypertensive patients [59]. In a sub-study of the Heart Outcomes Prevention Evaluation (HOPE) trial, patients receiving ramipril had a 32% reduction in the risk of any type of stroke and a 61% reduction in fatal stroke compared to the placebo group [34]. The benefits were observed in all patients, regardless of BP values. Beneficial effects were also obtained following treatment with ARBs.

In the LIFE (Losartan Intervention for Endpoint Reduction in Hypertension) trial, losartan administered to hypertensive patients with left ventricular hypertrophy resulted in a 24% reduction in major vascular events and a 21% reduction in stroke compared to placebo [60]. The analysis of a subgroup of patients with previous stroke within the SCOPE trial (Study on Cognition and Prognosis in the Elderly) showed that the administration of candesartan significantly reduced the rate of recurrent stroke and cardiovascular events [61]. Also, in the ACCES (Evolution of Acute Candesartan Cilexetil Therapy in Stroke Survivors) trial, candesartan administered to hypertensive patients on the first day of stroke, determined after one year, resulted in a significant reduction in mortality and cardiovascular events [62].

## 6. The Renin–Angiotensin–Aldosterone System and Heart Failure 

Secretion of AG II, stimulation of AT1 receptors, and release of aldosterone represent the main mechanisms involved in the pathogenesis of heart failure (HF). The main actions of aldosterone consist of cardiac effects (cardiac fibrosis), renal effects (sodium/water retention and potassium excretion), but also other effects (endothelial dysfunction).

There is a series of well-known studies in which there were patients with HF, and in which the administration of ACEIs and ARBs determined the decrease in cardiovascular mortality and the number of days of hospitalization [63,64,65,66]. There are also meta-analyses in which ACEI or ARB studies were included, which proved the effectiveness of the two drug classes in HF, especially in terms of reducing mortality [67,68].

Thus, in the CONSENSUS (Cooperative North Scandinavian Enalapril Study) [63] and SOLVD (Studies of Left Ventricular Dysfunction) [64] trials, it was demonstrated that the administration of ACEs, specifically enalapril, in patients with heart failure, increased survival and improved the NYHA class in patients with this condition. In the Val-HeFT trial (The Valsartan Heart Failure Trial), which included valsartan administered to patients with CHF, a statistically significant reduction of 13.3% in the risk of mortality and cardiovascular morbidity was determined [66]. The administration of candesartan in the CHARM trial (Candesartan in Heart Failure–assessment of reduction in mortality and morbidity) in patients with CHF who did not tolerate ACE inhibitors led to a 23% reduction in cardiovascular mortality and hospital days [65].

The latest ESC guidelines recommend ARBs in patients with reduced ejection fractions if they do not tolerate ACEIs or angiotensin receptor–neprilysin inhibitors. These drug classes reduce cardiovascular mortality and hospitalizations for HF [69].

## 7. The Renin–Angiotensin–Aldosterone System and Atrial Fibrillation

The RAAS is implicated in the occurrence of atrial fibrillation (AF) through several mechanisms, the most important of which would be the direct ones on the atrial structural and electrical properties and the indirect ones in the context of HF and arterial hypertension (important comorbidities of AF).

In arterial hypertension, the elevation of left atrial pressure and LV end-diastolic pressure occurs, which favors the development of AF [70]. In animal models with HF, ACEIs, and ARBs determined the regression of atrial remodeling and fibrosis and shortened the atrial effective refractory period [71]. At the same time, an analysis of human atrial myocytes taken from patients undergoing cardiac surgery reported increased tissue levels of ACE and AG II receptors in patients with AF compared to those in sinus rhythm [72].

The increase in the level of AG II activates the mechanisms of inflammation and fibrosis and the release of metalloproteinases with the excess production of collagen at the atrial level [73]. Genetic polymorphisms of ACE (ACE I/D polymorphism) and aldosterone synthase involved in the occurrence of AF have also been described [73]. At the same time, it is important to underline the fact that RAAS components are also synthesized at the local and atrial level.

The effects of ARBs respective to ACEIs in AF have been studied substantially. Thus, in the VALUE study that included hypertensive patients, the administration of valsartan was accompanied by a decrease in the risk of AF by 24% in the first 3 years, and 16% after 6 years [74].

In a systematic review and meta-analysis of 26 randomized clinical trials, it was shown that RAAS blockade had an important role in the prophylaxis of AF in patients with HF, causing a 24% reduction in the risk of its occurrence [75]. Other data show that RAAS blockade resulted in a 34% reduction in progression to permanent AF [73].

In a meta-analysis published in 2015, it was demonstrated that the use of ACEIs and ARBs in comparison with beta-blockers, calcium blockers, or diuretics, prevented the occurrence of AF in patients with systolic HF and those with arterial hypertension [73].

The role of anti-aldosteronics in the development of AF is also discussed. Thus, eplerenone was beneficial in maintaining sinus rhythm after catheter ablation in patients with permanent AF [76]. A very recent meta-analysis shows that the administration of anti-aldosteronics in patients with cardiovascular disease or at risk of developing cardiovascular disease is equally effective in preventing cardiovascular events in patients with and without HF and most likely retains its effectiveness regardless of the presence of AF [77]. They have demonstrated moderate effectiveness in preventing the onset or recurrence of AF episodes [77].

The 2023 ESH Guidelines for the management of arterial hypertension emphasize the importance of RAAS blockers, along with beta-blockers, in the prevention of AF recurrences in hypertensive patients [7].

## 8. Conclusions

RAAS plays a primary role in the pathogenesis of atherosclerosis, risk factors, and cardiovascular diseases, exerting significant effects throughout the entire cardiovascular continuum.

## Data Availability

No new data were created or analyzed in this study. Data sharing is not applicable to this article.
